# Uncovering functional signature in neural systems via random matrix theory

**DOI:** 10.1371/journal.pcbi.1006934

**Published:** 2019-05-01

**Authors:** Assaf Almog, M. Renate Buijink, Ori Roethler, Stephan Michel, Johanna H. Meijer, Jos H. T. Rohling, Diego Garlaschelli

**Affiliations:** 1 The Big Data Lab, Department of Industrial Engineering, Tel-Aviv University, Ramat Aviv, Israel; 2 Instituut-Lorentz for Theoretical Physics, Leiden Institute of Physics, University of Leiden, Leiden, The Netherlands; 3 Laboratory for Neurophysiology, Department of Molecular Cell Biology, Leiden University Medical Center, Leiden, The Netherlands; 4 IMT School for Advanced Studies, Lucca, Italy; Radboud Universiteit Nijmegen, NETHERLANDS

## Abstract

Neural systems are organized in a modular way, serving multiple functionalities. This multiplicity requires that both positive (e.g. excitatory, phase-coherent) and negative (e.g. inhibitory, phase-opposing) interactions take place across brain modules. Unfortunately, most methods to detect modules from time series either neglect or convert to positive, any measured negative correlation. This may leave a significant part of the sign-dependent functional structure undetected. Here we present a novel method, based on random matrix theory, for the identification of sign-dependent modules in the brain. Our method filters out both local (unit-specific) noise and global (system-wide) dependencies that typically obfuscate the presence of such structure. The method is guaranteed to identify an optimally contrasted functional ‘signature’, i.e. a partition into modules that are positively correlated internally and negatively correlated across. The method is purely data-driven, does not use any arbitrary threshold or network projection, and outputs only statistically significant structure. In measurements of neuronal gene expression in the biological clock of mice, the method systematically uncovers two otherwise undetectable, negatively correlated modules whose relative size and mutual interaction strength are found to depend on photoperiod.

## Author Summary

In recent years an increasing number of studies demonstrate that functional organization of the brain has a vital importance in the manifestation of diseases and aging processes. This functional structure is composed of modules sharing similar dynamics, in order to serve multiple functionalities. Here we present a novel method, based on random matrix theory, for the identification of functional modules in the brain. Our approach overcomes known inherit methodological limitations of current methods, breaking the resolution limits and resolves a cell to cell functional networks. Moreover, the results represent a great potential for detecting hidden functional synchronization and de-synchronization in brain networks, which play a major role in the occurrence of epilepsy, Parkinson’s disease, and schizophrenia.

This is a *PLOS Computational Biology* Methods paper.

## Introduction

Understanding how billions of neurons collectively self-organise into a functionally ordered brain able to coordinate a variety of neural, cognitive and bodily processes is probably the most fundamental quest in neuroscience. Over the last decades, evidence has accumulated that the functional organisation of the brain is modular and hierarchical [1]. This means that the brain appears to be partitioned into mesoscopic ‘functional modules’ where each module is composed of neurons with a relatively similar dynamical activity, while different modules are comparatively less related to each other. Each such module may also contain submodules hierarchically nested within it.

Reliably identifying functional modules is a nontrivial task because of their irreducibility to contiguous anatomical regions defined *a priori* and/or to local neighbourhoods in the underlying structural network of neuron-to-neuron anatomical connections [2]. Indeed, while on the one hand functional modules partly reflect the local brain anatomy, on the other hand major deviations between functional and structural networks are observed. One key example is the distinctive ‘long-range’ left-right splitting of some functional modules: often, a single module is found to be composed of two or more spatially non-contiguous populations of neurons, located in possibly distant (sometimes symmetric, sometimes asymmetric [3]) regions in the left-right direction [4, 5]. As an opposite example, an anatomically well-defined brain region can be functionally heterogeneous [6, 7] and sometimes even display anti-correlation between the activity of some of its parts [8, 9]. These examples indicate the lack of a one-to-one correspondence between structural and functional modules, showing that it is in general impossible to infer the latter purely from spatial information. Indeed, it is expected that the mapping between functional and structural networks is many-to-one, thus allowing a diversity of functions to arise from a common neuronal anatomy [2]. On top of this, both structural and functional brain networks are characterized by *plasticity*, i.e. possibility of temporal rearrangements, but at typically different spatial and temporal scales.

**Fig 1:**
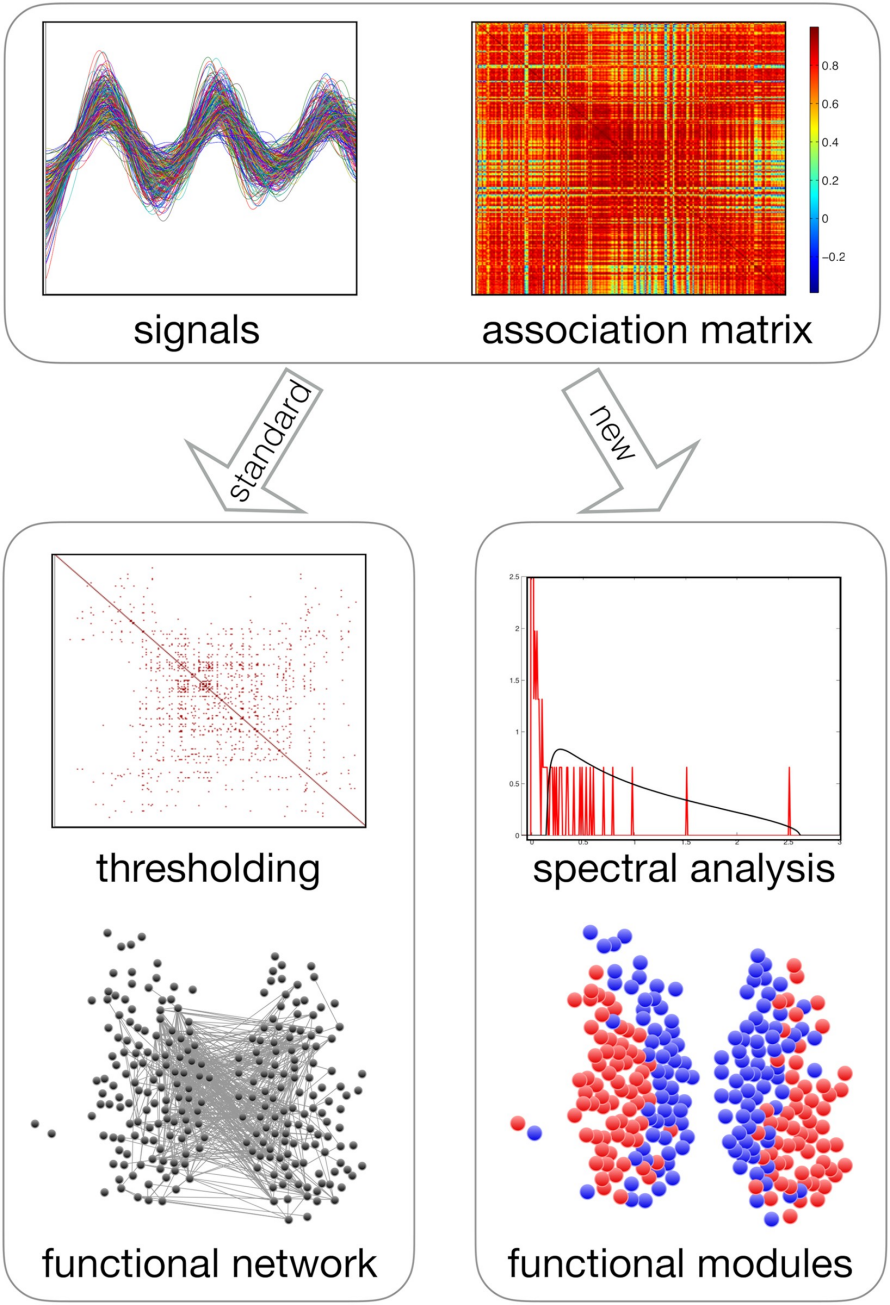
Illustration of the procedure of functional module identification from time series data (top) in the standard approach (bottom left) and in our method (bottom right). In this example (our empirical data), we can see that the signals share a very strong common periodic trend, which results in very high correlation values. In the standard approach, an arbitrary threshold is defined and the original matrix is projected onto a functional network. This comes at the price of discarding the majority of the data, most notably the negative correlations, and makes the output threshold-dependent. Moreover, modules are searched for in the projected network using null models that are valid for graphs with independent edges, but not for correlation matrices. We should note that there are recent approaches, which will be discussed in the next section, that do not discard negative correlation. In our method, we compare the empirical correlation spectrum against a null model specifically tailored for correlation matrices. This produces a filtered correlation matrix that is subsequently searched for modules. These modules are guaranteed to be statistically significant, noise-free, overall positively correlated internally and overall negatively correlated across. By directly producing a partition of the original time series into modules, our method bypasses the functional network projection, avoiding the use of a threshold.

Precisely because they cannot be reduced to ‘spatially obvious’ brain regions, functional modules must entail an emergent, non-structural level of neural organisation which can only be investigated via the explicit analysis of time series of activity of individual neurons or, at a more coarse-grained level, regions of interest (ROIs). More specifically, recordings of multiple time series are normally used to construct an association (e.g. cross-correlation, mutual information, etc.) matrix capturing the mutual relations between pairs of ROIs (see Fig 1). Next, the matrix can be analysed in different ways to detect the presence of functional dependencies or structure in the system.

Importantly, these dependencies can be positive (+) or negative (−), leading to measured correlation or anti-correlation. For instance, synaptic interactions between neurons will influence their mutual phases and lead to different states of synchronization in a brain circuit. The degree of synchronization (+) versus desynchronization (−) is important for neural function and a disturbance in this balance can contribute to neurological disorders. In the paradigmatic example of the central mammalian clock situated in the *suprachiasmatic nucleus* (SCN) of the hypothalamus, the state of synchronization of neurons can influence responses of the circadian system to light and is actually used to encode seasonal changes in day length. It has been suggested that inhibitory (−) as well as excitatory (+) neuronal interactions will contribute to the phase differences observed under different photoperiods [10, 11]. The balance between excitatory and inhibitory activity (E/I balance), which is a hallmark of healthy network performance, can actually change with photoperiod [12].

The motivation for the present paper is the expectation that, in the brain and in possibly many other biological networks as well, the presence of both positive and negative interactions should have a significant impact on how the modular functional organization is both mathematically defined and empirically identified. For instance, even within a functionally homogeneous region there may be negatively correlated substructures arising from the need to create and/or modulate the internal mutual phase relationships. Similarly, across two functionally distinct modules there may be a need for dependencies of both negative and positive sign, depending on whether the two functions should inhibit or enhance each other. Consequently, we stress that a proper definition of functional modules should take the sign of the defining correlations into serious account and tools should be devised to reliably identify such sign-dependent structure from time series data. This is crucial in order to map how function is distributed across the modular brain landscape and to properly constrain models of the underlying neural dynamics.

In this paper, we argue that the available approaches to the theoretical definition and empirical detection of functional modules treat negative dependencies in essentially unsatisfactory ways. On one hand, most techniques either entirely dismiss negative values [13], or turn them into positive ones [14], thereby using no information about the sign of the dependency. On the other hand, the few methods that do take negative correlations into account use (null) models that treat all pairwise correlation coefficients as statistically independent entities, thus violating important structural properties of correlation matrices. Other popular approaches like Principal Component Analysis (PCA) or Independent Component Analysis (ICA) look for *independent*, rather than anticorrelated, components, thus serving a different purpose. Moreover, most of these approaches fail to provide a stastistical validation of the modules identified, and are therefore prone to misidentification due to the presence of both ROI-specific noise and brain-wide common trends obfuscating the underlying mesoscopic modular patterns.

Here, we propose a novel method that targets specifically the positive and negative interactions in brain data and filters the underlying noise and common trends using an appropriate null model based on Random Matrix Theory (RMT). Our approach generalizes a recent community detection method tailored for correlation matrices [15, 16], originally formulated for financial time series that have an inherently random and non-periodic pattern, and extends it to the case where arbitrarily structured temporal trends are allowed. We also pay specific attention to the fact that noise and global trends have a previously overlooked coupled effect on the spectrum of correlations, and we rigorously correct for this coupling. Technically, the method makes use of a modified Wishart ensemble of random correlation matrices constructed using precisely the same common trend and expected noise level as the empirical time series, but under the null hypothesis that no modular organization is present. This ensemble serves as a natural, reliable and more appropriate null model for correlation matrices arising in brain research. A comparison between empirical and null correlation matrices reveals the functional modules present in the data and by construction absent in the model.

The resulting method is threshold-free and does not require the arbitrary projection onto a network (see Fig 1). Moreover, in contrast with most of the current approaches, it is designed to yield an optimally sign-contrasted structure, where positive interactions are clustered inside the modules and negative values are expelled across modules. We call the resulting optimized structure the *functional signature* of the system. This structure is composed of functional modules whose overall internal correlation is guaranteed to be positive and whose overall mutual correlation is guaranteed to be negative. The method only outputs statistically significant structure, if present. We should stress that in any stage of the process there are no presumptions about the output of the method (such as a predefined number or size of modules) and the results are completely and non-parametrically driven by the data themselves. If needed, the method can be used iteratively to detect sub-modules hierarchically nested within modules.

Besides formulating the method, we apply it to the analysis of the aforementioned SCN, which is responsible for regulating the circadian rhythms of physiology and behaviour in mammals. We chose the SCN of mice because of its relatively small size (ca 20,000 neurons) and high degree of functional plasticity. Single SCN neurons are capable of generating circadian rhythms in, amongst others, gene expression and electrical activity. The phase differences between the cells can vary with changes in the environment, such as different photoperiods or prolonged light exposure, or with an attenuation of the degree of coupling between the neurons as seen in aging or disease. This makes the SCN an optimal case study for a dynamic network of neurons with different internal oscillations, mechanistically coupled to E/I processes.

We show how our method can be used to reliably search the SCN for sign-dependent functional modules reflecting the phase ordering of oscillating cell populations, based on both strength and sign of their coupling interactions. We use samples taken from mice that were subjected to different photoperiods. The method identifies two otherwise undetectable clusters of functionally connected SCN neurons that have a strong resemblance to a known core/shell distinction [17] and that have never been found before without the use of prior knowledge. Importantly, we are able to detect physiological differences present in different photoperiods in the functional signature of the two clusters. We find that the sizes of the two modules change with photoperiod as the result of a majority of neurons remaining in the same module irrespective of photoperiod, and a minority alternating between the two modules at their interface. This finding highlights a possible population of alternating neurons responbile for the functional plasticity required for adjustment to photoperiod and circadian modulation.

## Results

### Limitations to overcome in the identification of sign-dependent functional modules

Our approach aims at overcoming various limitations of the existing methods. It is therefore convenient to briefly mention these limitations in order to gradually introduce some of the defining elements of our method.

First, we want to avoid the use of thresholds on the entries of the correlation matrix. Indeed, most approaches identify functional modules via the introduction of a threshold used to project the original correlation matrix into a network (see Fig 1) [18, 19]. On this network, various graph-theoretic quantities can be measured to identify modules in terms of e.g. connected components [20], rich clubs [21], *k*-cores [22] or communities [23]. The well known limitations of this approach are the uncontrolled information loss induced by discarding some of the observations, the complete arbitrariness of the choice of the threshold value, and the resulting unavoidable threshold-dependence of the output [24]. Moreover, since thresholds are introduced to project the original matrix into a sparse network, and since the number of negative entries in such matrix is usually smaller than that of positive ones, this procedure essentially imposes a positive threshold, thereby completely disregarding all the negative correlations.

Second, we want to avoid turning the negative correlations into positive ones. Based on the (correct) consideration that negative correlations indicate functional dependency (rather than no dependency), many approaches aim at exploiting both positive and negative values as cohesive interactions in the definition of functional modules. To this end, they take e.g. the absolute value or the square of the original correlations. However in this way the negative correlations are treated just like the positive ones, making it impossible for the output modules to encode any information about the original sign of the functional dependencies. We instead believe that the sign should be retained and used as a repulsive interaction in the definition of modules, with the understanding that the latter should not be interpreted as functionally independent of each other, but rather as dependent sub-modules in mutual anticorrelation, possibly nested within larger modules that may eventually be functionally unrelated.

Third, we want to avoid the ‘merging bias’ that affects even the few remaining methods that do preserve the sign of correlations in the definition of modules [14, 25, 26]. These methods are adaptations of the so-called ‘modularity maximization’ techniques introduced in the literature about community detection in networks and targeted at finding groups of nodes that are more densely connected internally, and less densely connected across, than expected under a random null model [23]. The main null models for networks have statistically independent links, i.e. a link can be placed between any two nodes without affecting the probability of placing links elsewhere in the network. The methods that generalize these null models to correlation matrices extend them in the direction of allowing links with both positive and negative weight, but unfortunately retain the assumption of independent matrix entries [14, 25, 26]. While justified for networks, this assumption becomes incorrect for correlation matrices, whose entries are subject to basic ‘metric’ properties that make them depend on each other [15]. For instance, negative triangular relationships of the type *C*_*i*,*j*_ < 0, *C*_*j*,*k*_ < 0, *C*_*k*,*i*_ < 0 are in general very rare in empirical correlation matrices (and become impossible if *C*_*i*,*j*_ = *C*_*j*,*k*_ = *C*_*k*,*i*_ = −1), while they are much more likely in a null model with independent entries. This effectively creates the systematic bias of erroneously interpreting the absence or scarcity of such negative triangles in the data as strong statistical evidence for the nodes *i*, *j* and *k* being ‘attracted’ to each other. As a net result, the three nodes are likely to be merged in the same module (hence the merging bias), although their mutual anticorrelation represents statistical evidence that they should in fact belong to three separate modules.

Fourth, we want to avoid misidentification due to the presence of common trends across all ROIs in the sample. Indeed, depending on the spatial and temporal resolution of the data, experimental time series may contain a multitude of periodic or systematic trends at different frequencies (e.g. heartbeat, breathing, circadian rhythms) that impart an overall positive correlation to all or several ROIs, without actually representing any real functional relatedness among the ROIs themselves. One of the side effects of such ‘global mode’ is a reduction of the detectability of the underlying modular structure. Certain techniques aim at solving this problem by preliminary subtraction of the measured average trend from each time series separately (thus effectively removing the global mode), and then calculating the resulting correlation matrix. Along these lines, methods like Bazzi et al [27] proposed a null model in which the elements are correlated at some baseline level, where the amplitude of this level is determined by a tunable parameter. These procedures have been criticized because they tend to generate both positive and negative correlations by construction, with no guarantee that the corresponding signs represent a true signature of functional modularity, e.g. even if the original time series were all independent and their increments relative to the average trend were merely due to chance or noise.

Fifth, and connected to the point above, we want to accurately characterize the level of noise in the data. This point is connected to many of the points above. For instance, being able to separate noise from information would allow us to avoid the use of arbitrary thresholds, discriminate between true and random modularity, and arrive at a safer definition of modules based on trends relative to the global one, thus enhancing the detectability of functional substructure.

### A random matrix null model for correlation matrices of neural activity

We are now ready to introduce our method which is designed in order to avoid the limitations described above.

Given an empirical correlation matrix constructed from multiple time series of neuronal activity, our method looks for functional modules upon removing the joint effects of noise in the data and of common temporal trends, as both features may obfuscate the empirical identification of possible underlying substructure. For this task the method first introduces a null model that serves as a random benchmark, thus accurately highlighting the non-random modular patterns in the empirical correlation matrix. This improved null model, based on random matrix theory, takes into account cell to cell variability and does not require the unrealistic assumption that the time series are stationary. Therefore we can allow for any temporal modulation (see section Materials and methods), both in individual time series and in their resulting common trend. This is very important, given the strongly time-dependent nature of functional brain data in general, and of our time-modulated oscillating signals in particular. So, even if the calculation and interpretation of correlation matrices usually assumes stationarity, here we can statistically treat correlation matrices calculated from nonstationary data as well.

**Fig 2:**
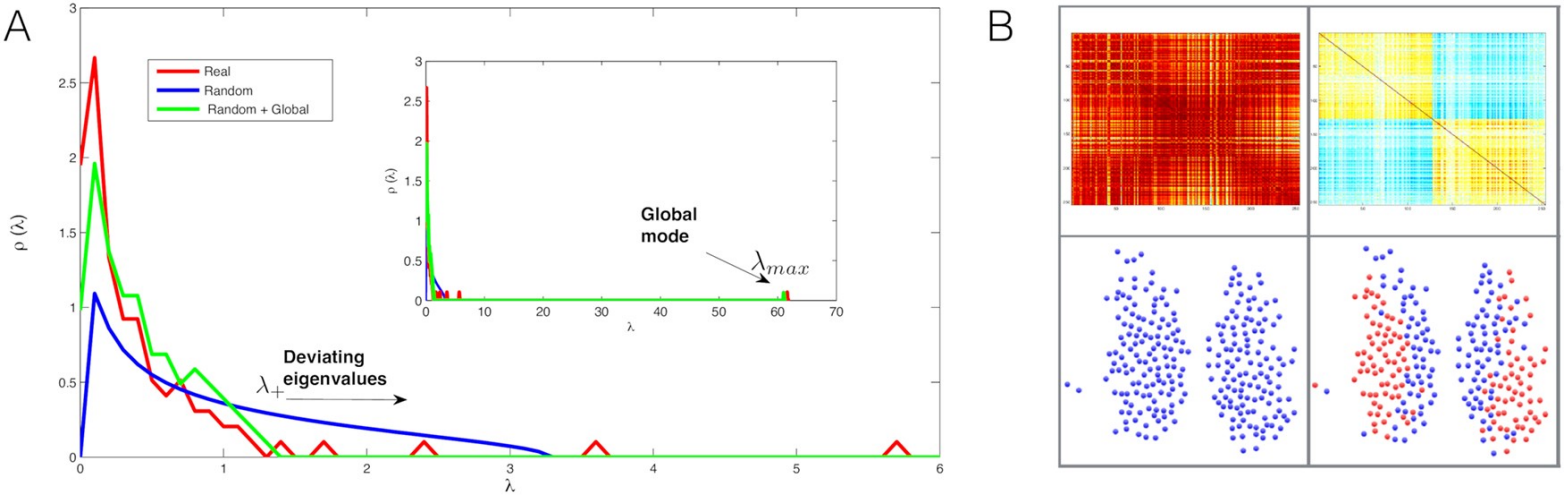
(A) Empirical eigenvalue density versus calculated eigenvalue density for the two random models. (B) The community structure of the SCN as resolved by our method. On the left, is the community structure detected by random model without filtering the global mode (Random). On the right, is the community structure detected by random model once the global mode is filtered (Random + Global). In the bottom panels are the partitions detected, where each community is marked with a different colour. In the top panels are the corresponding resolved filtered correlation matrices displaying the resolved structure as a block matrix.

The first step is an exact calculation of the combined, undesired effects of noise and common trends on the density of eigenvalues *ρ*(λ) of a theoretical cross-correlation matrix. This step corresponds to the definition of a null model for a correlation matrix without modular patterns, but with a noise level calibrated to the observed one and with a global trend that exactly follows the one in the empirical time series. The output of this first step is illustrated in Fig 2A. The density of eigenvalues, which is calculated exactly in the null model [see section Materials and methods], features one largest eigenvalue λ_*max*_ due to the global trend, plus a “random bulk” extending between a minimum (λ_−_) and a maximum (λ_+_) eigenvalue.

The second step is a filtering of the original correlation matrix via the identification of the empirical eigenvalues that deviate, in a statistically significant manner, from the ones predicted by the module-free null model. In practice, this reduces to the selection of the empirical eigenvalues that are found in the range (λ_+_, λ_*max*_). A crucial result in this study, overlooked in previous analyses [15], is a precise calculation of λ_+_ showing that the higher λ_*max*_, the lower λ_+_. The fact that the values of λ_*max*_ and λ_+_ depend on each other is a proof that noise and global trends jointly affect the features of the expected eigenvalue density of the correlation matrix. Our calculation of λ_+_ allows us to recover statistically significant features of the empirical correlation matrix that would otherwise be incorrectly classified as noise. Looking again at Fig 2, we indeed see the presence of eigenvalues in the empirical spectrum (red) that deviate from our adjusted null model (green) and include eigenvalues that would be incorrectly classified as noisy if λ_+_ were not corrected for λ_*max*_ (blue). This step is completed by the selection of the eigencomponent of the correlation matrix associated with the deviating eigenvalues. The resulting, cleaned component of the original matrix contains statistically significant, noise- and trend-filtered information about the presence of functional modules.

### Detecting functional signature in neural systems

Once the original correlation matrix has been filtered by the null model, only the statistically significant dependencies are guaranteed to remain in the matrix. At this point our aim is the identification of functional modules that are positively correlated internally and negatively correlated externally. This can be transformed into an optimization problem. We employ community-detection techniques that take the filtered correlation matrix as input and return the optimized partition of the system into functional modules. The optimized partition will tend to place the positive dependencies (correlation) inside the clusters while expelling the negative dependencies (anti-correlation) across the clusters. We should stress that, by construction, the emergent functional structure will be detectable only if it is statistically significant. Moreover, the number of detected clusters is not defined *a priori*, and is found automatically by the method itself.

It should be noted that, while the use of information contained in the eigenvectors of the largest eigenvalues is common to other methods (such as Principal Component Analysis and it generalization, aka Independent Component Analysis [28, 29]) as well, our approach distinguishes itself from these approaches in various respects. First, those methods look for the *independent* components in which the orginal signal can be optimally decomposed, while our aim is to pinpoint the *anticorrelated* groups of units. Second, our iterative optimization procedure reformulated for correlation matrices guarantees that the final output is maximally contrasted in terms of the signs of the detected modules. Finally, the other approaches focus on the strongest eigenvalues but do not implement a null model, tailored to capture both local noise and global trends, to assess which of the eigenvalues are informative and which are noisy. Indeed, in ICA the desired number of components has to be specified by the user, whereas in our method the optimal number of modules is given as output by the algorithm.

**Fig 3:**
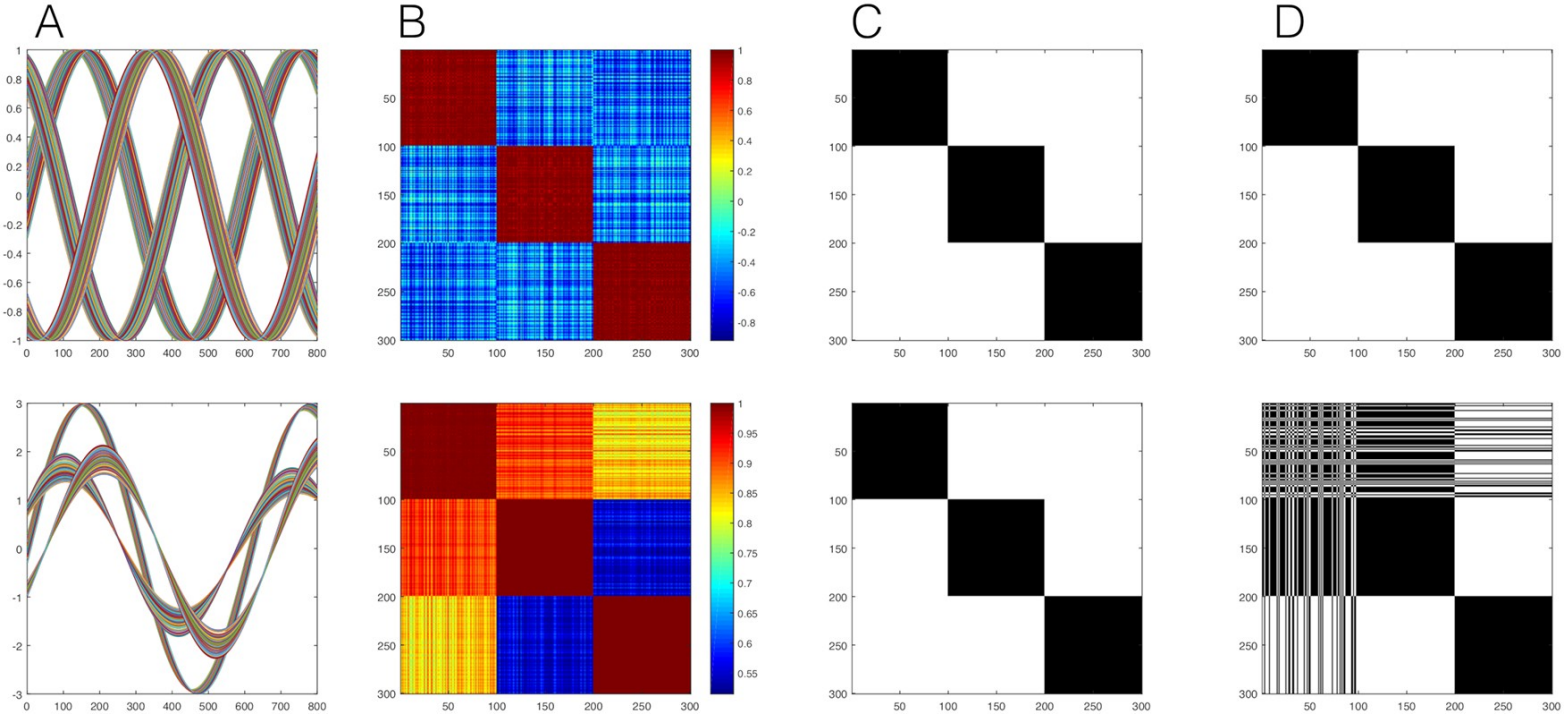
Illustration of misidentification due to the presence of common trends in a comparison between the method by Rubinov and Sporns [14] (based on a null model with independent entries of the correlation matrix) and our alternative approach (based on the more appropriate null model with dependent entries constructed from random matrix theory). (A top) 300 synthetically generated time series in a system with 3 modules, each containing 100 oscillating series with random phases, (A bottom) 300 synthetically generated time series as before, with a strong global periodic signal. (B) the corresponding correlation matrix, top and bottom accordingly, showing a clear block structure. The output of our method (C) and the Rubinov-Sporns method (D) in terms of likelihood matrices indicating the frequency with which two neurons are found in the same community in 1000 runs of both methods. We can see that the Rubinov-Sporns method is able to correctly separates the 3 modules in the more straightforward case of clear positive and negative correlation (D top). In the more complex case where the correlation are obscured by a strong common trend the Rubinov-Sporns method merges the first module with the other two clusters. Using the proper null model our method is able to correctly separates the 3 modules in both cases (C).

By using an appropriate null model that takes into account the presence of strong global trends, our method avoids the misidentification due to common trends and merging bias of other methods described above. To illustrate this, in Fig 3 we show a synthetic sample with 300 oscillating signals divided into 3 main groups, in each of which 100 signals are randomly assigned different phases around a ‘master signal’ (top). We also consider the same exact system with strong (periodic) global trend, which obscure the positive and negative correlations (bottom). We can clearly see that due to the differences in phase between the groups, the relations between different groups become negative (anti-correlation) in sample one (top), however, in the system with the global trend all of the correlations are shifted to positive values (bottom). We then process the correlation matrix with the (independent-entries) method proposed in [14] and with our method. While our method is able to cluster the 3 groups perfectly in both cases, the general modularity method clusters the modules correctly only in simple case where no global trends are present.

### Uncovering the hidden functional signature of the SCN

The brain region we apply our method to is the suprachiasmatic nucleus (SCN), located in the hypothalamus in the brain, and recognized as the site of the central circadian clock in mammals. This clock is important for the regulation of our daily and seasonal rhythms. It has been shown that the neuronal network organization of the SCN changes in different photoperiods [30], however, the mechanisms behind these changes are still elusive. Furthermore, only a subset of neurons within the SCN network are directly responsive to light [31], which poses the question how encoding for seasonally changing day length is achieved in the SCN network. The SCN is a prototypical example of a brain structure for which resolving functional organization is challenging for the reasons outlined above: it consists of about 20000 neurons that are spatially close (total size of 1 *mm*^3^—so, structurally speaking, these neurons form a single densely connected cluster, whose only anatomical substructure is a left-right split into two lobes) while at the same time displaying a high variability in terms of the signals of the constituent neurons.

Currently, brain networks are most often derived from data acquisition techniques that do not have the possibility to perform recordings at the single cell level. Techniques such as (functional) Magnetic Resonance Imaging ((f)MRI), Electroencephalography (EEG) or Magnetoencephalography (MEG) use brain regions as nodes in the network and statistical associations of regional/sensor temporal activity as edges. We investigate the SCN network at the micro-scale where nodes are single cells and edges are functional connections between the cells. We use single-neuron data on gene expression of a clock gene *period2* in the SCN. The data were sampled every hour for at least three days by means of a bioluminescence reporter PER2::LUC.

**Fig 4:**
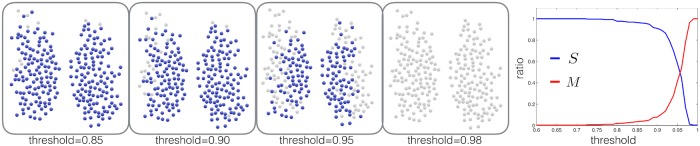
The community structure of the SCN as resolved by a standard threshold approach. On the left, we plot the community structure, resolved by the standard method, for different thresholds. In blue are the nodes that belong to the large cluster, while in gray are isolated nodes (’communities’ that only contain one node). In the right panel, we plot the fraction of nodes in the largest connected component *S* in blue, and the fraction of communities detected *M* in red.

We first perform a standard analysis based on the mainstream method [see Fig 1] for detecting communities via functional networks. This is a useful reference as a comparison with our own method. In Fig 4 we present the community structure, resolved by the standard method, for different thresholds. In blue are the nodes that belong to the large cluster, while in gray are isolated nodes (communities that only contain one node). In the right panel, we plot the fraction of nodes in the largest connected component $S=\frac{LCC}{N}$ in blue, and the fraction of communities detected $M=\frac{Communities}{N}$ in red. It is evident that applying different thresholds essentially detaches isolated nodes from the large cluster, and there is no optimal value for the threshold. Therefore, the standard method can only observe a “radial gradient” of connectivity, and there is no sense of multiple communities of neurons, which is one of the signatures of functional as opposed to structural connectivity. This poor performance of the method is a known limitation when applied to very dense networks.

Our method detects mostly two communities which coincide with the core and shell distinction within the SCN [17]. The core of the SCN receives light input and adjusts quickly to changing light schemes, while the shell of the SCN lags behind [32]. Mostly the core-shell distinction of the SCN is interpreted as a distinction between the ventrolateral and the dorsomedial part of the SCN, which is predominantly based on anatomical data [33]. In this study the two clusters that were found were more dorsolaterally and ventromedially located, and while it is based on functional data this may differ from known anatomical distinctions. Furthermore, the SCN is much more heterogeneous when looked at cellular phenotype or gene expression [6, 34]. The anatomical loci do not necessarily delineate the phenotypical SCN regions very precisely, which implies that functionally, the core-shell distinction is less clearly defined and may differ from the described anatomical division (see also [17]).

**Fig 5:**
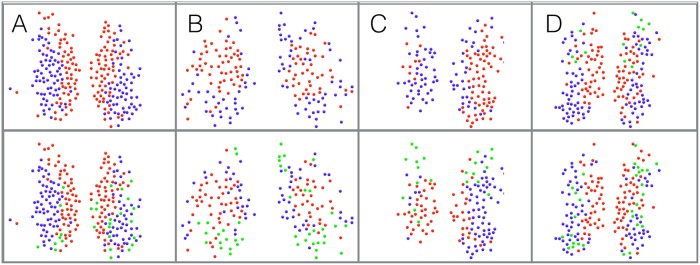
The community structure of the SCN for 4 different samples (A,B,C,D) as resolved by our method (top) and by the Rubinov and Sporns [14] approach. The panels represents the partitions detected, where each community is marked with a different colour. We can see that the Rubinov Sporns method shares a good resemblance to the clear core periphery structure that is detected by our method. In contrast to the random matrix approach, the signed Leuven consistently detects a third module, in a different location at each time.

Next, we perform the analysis using the method presented in Rubinov& Sporns(2011) [14], which uses a modified modularity matrix to incorporate signed matrices. Since the method does not require a threshold parameter and using all the data entries in the correlation matrix to resolve the community structure, we anticipate a better performance than the standard threshold procedure. In Fig 5 we plot the community structure of four different samples (A,B,C,D) as resolved by the two methods. The panels represents the partitions detected, where each community is marked with a different colour. Strikingly, the signed Leuven method community structure is very comparable to the clear core periphery structure that is detected with the random matrix approach. However, it also detects with high consistency three communities, when the third community is changing in size and location for each sample. The differences in the structures might result from the improve ability of the random matrix approach to filter common trends, as seen in Fig 3. Nevertheless, the presence of the general core periphery pattern is reinforced by both of the methods.

**Fig 6:**
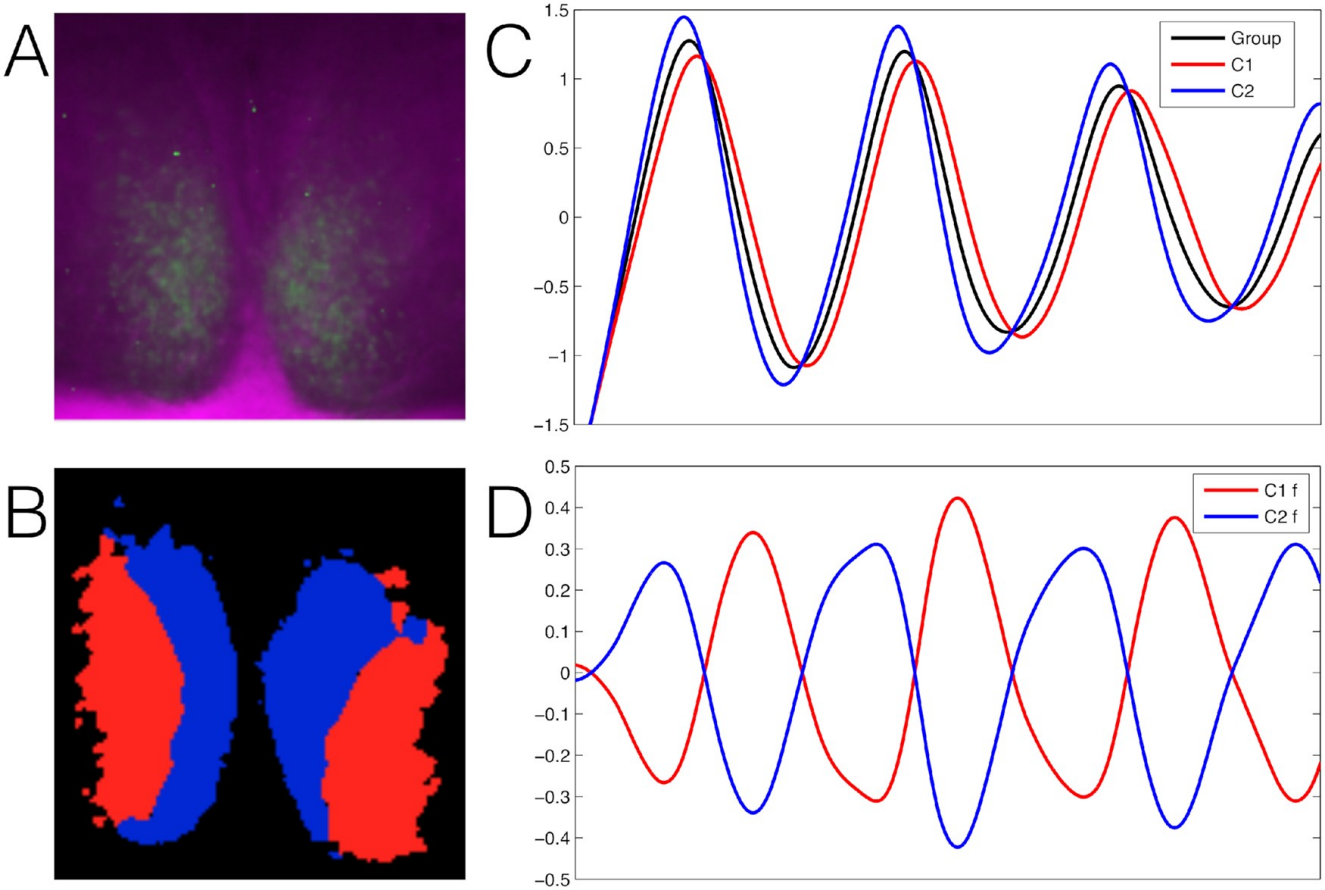
(A) The bioluminescence image of one SCN sample. (B) The plotted average partition over all the samples. (C) The plotted average signal of the whole system (in black) versus the mean signals of the two detected communities (in red and blue) for one SCN sample. (D) The plotted average residual signals of the two communities of one SCN sample, once the global signal is subtracted.

Regional analysis of the SCN using functional time series has been performed by other groups. Evans and co-workers used a similar approach to identify single-cell-like regions of interest, but did not use clustering algorithms and chose the regions by hand [35]. Silver and co-workers also used regions of interest, called superpixels, but these were not necessarily identified as single-cells. Based on these superpixels they used threshold methods to identify regional differences in the SCN [36, 37]. Abel and co-workers applied a threshold method based on mutual information on single-cell-like regions of interest [38]. These approaches encounter similar problems as described in this paper when using the threshold method: they only find one cluster (in the core, or ventral part) and many non-clustered cell-like ROIs (in the shell or dorsal part). Our results presented here are in line with the regional division of the SCN proposed in these studies, but we were able to identify both the core and shell clusters. To visualize the general community structure we plot the bioluminescence image of one SCN sample with the resolved average partition average partition over all the samples (Fig 6A and 6B). We also plot the average signal of each community to observe the optimized anti-correlation pattern (Fig 6C and 6D), which corresponds to the functional signature of the SCN. Furthermore, our approach is able to identify the two clusters in different experimental conditions, ranging from summer conditions (long days, short nights: LD 16h:8h) to winter conditions (short days, long nights: LD 8h:16h). On the contrary, Evans and co-workers identified changes occuring in the organization of the SCN, where the two regions similar to our clusters were found, only for very long day conditions (LD 20h:4h) [39].

**Fig 7:**
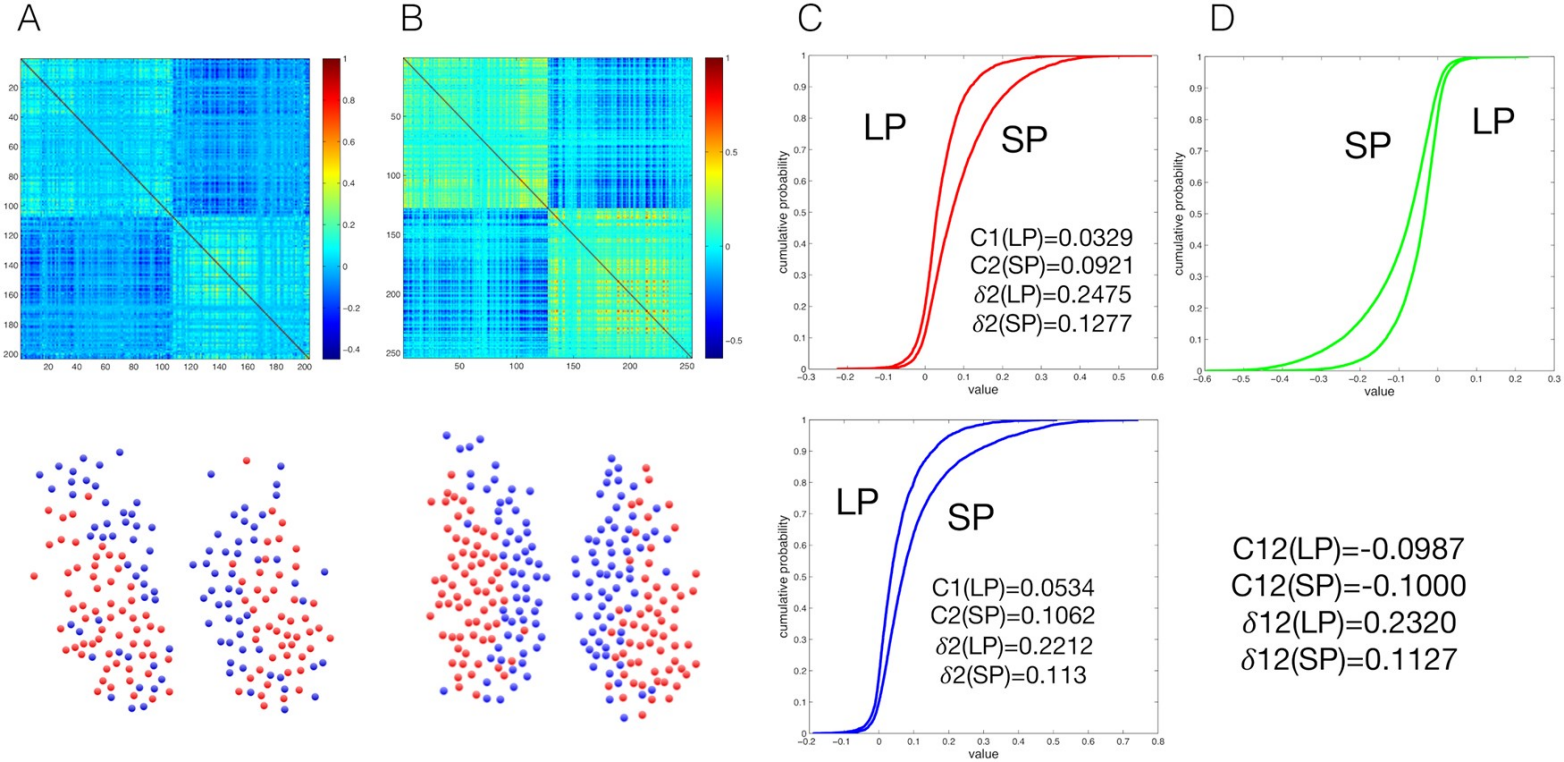
(A) The resolved functional signature and modules structure of a long photoperiod (LP, L16D8) sample. (B) The resolved functional signature and modules structure of a short photoperiod (SP, L8D16). (C) Cluster analysis: plotting the cumulative distribution of the dependencies within the two detected clusters, comparing the two different photoperiods. The upper graph shows cluster 1 and the lower graph cluster 2. *C* represents the measured averaged correlation of a cluster, and $\delta \equiv \frac{N_{-}}{N_{+}}$ is defined as the contrast ratio of a cluster, measuring the ratio of negative dependencies versus positive dependencies. (D) Inter-cluster analysis: plotting the cumulative distribution of the external dependencies between the two detected clusters, comparing the two different photoperiods. *C* represents the measured averaged anti-correlation between clusters, and $\delta \equiv \frac{N_{+}}{N_{-}}$ is defined as the contrast ratio of a cluster, measuring the ratio of positive dependencies versus negative dependencies.

As a next step we analyzed the values in the functional signatures and compared those between different photoperiods that the animals have been subjected to. With this step we reveal the dynamics within the population of neurons in the clusters and between the clusters. As the cluster-partition is based on the functional signature, we will now investigate the values within and between the clusters, exploring the inner and outer level of correlation. This extra information links physiological properties of the SCN to the functional signature found in the data. We measure the average residual correlation within each cluster detected by our method and we plot the community distribution of the measured values (Fig 7A and 7B). We then identify the cumulative probabilty of the values in the clusters and we see that in short photoperiods the average values are much higher than in long photoperiods (Fig 7C). This means that the correlation within the clusters is significantly higher in short photoperiods than in long photoperiods. When we examine the values between the clusters, we see that the average value is lower in short versus long photoperiod, meaning that the clusters are less correlated in short phtoperiods (Fig 7D). These results connect directly to previous results in physiological properties as described in [7] and is supported in other papers [30, 40]. Thus, we show that the hidden functional representation reveals the phase ordering of oscillating cell populations caused by physiological properties of the SCN.

## Discussion

Our method reveals hidden functional dependencies that are obfuscated by the presence of a global mode in the neuronal gene expression, which imparts an overall positive correlation. This problem becomes particularly evident when searching for functional structure in neuronal systems where the global signal is very strong, making the identification of functional modules very challenging. Our method is able to deal with the effects of noise and common global trends in the original data in a robust manner. In fact, we have shown that the effects of noise and those of the global signal are coupled, as their signatures in the spectrum of the correlation matrix depend on each other.

We found a distinctive left-right functional symmetry with core-shell features in the SCN. This structure reveals non-contiguous regions that display strongly synchronized activity, despite being at a relatively large distance from each other, similar to [4]. Remarkably, here we detect this functional symmetry on a micro-scale level where nodes are single cells. In this respect, it is important to notice that while the traditional threshold method applied to the SCN resolves only a radial gradient of functional connectivity that closely mirrors the anatomical proximity of neurons without singling out any modularity or boundary, our method systematically reveals two sharp modules, a ventral core and a dorsal periphery. These modules feature distinct signatures of functional (as opposed to structural) connectivity, namely left-right symmetry, spatial non-contiguity, and almost perfect dynamical anti-correlation once the global SCN-wide signal is filtered out. The left and right shell regions of the SCN, despite being spatially disconnected into two non-contiguous regions, are functionally joined into a single module. These symmetrical structures in the SCN raise important questions with respect to the mechanism in the system, and can possibly be explored in the future.

The ability to exploit all the information from the correlation matrix, i.e. both the negative and the positive dependencies (correlation and anti-correlation), in order to detect the functional modules is very powerful. The strength of our method is to detect communal phase differences in neuronal networks by analysing time series data without using any presumptions or threshold definitions. Phase differences and phase adjustments in neuronal networks are an key feature for physiological function and can be used to define the functional state of a network in health and disease. Our method allows the identification of synchronized clusters of cells. Synchronization within a neuronal network was suggested to play a major role in the occurrence of epilepsy [41, 42], Parkinsons disease [43, 44] and schizophrenia [45, 46]. It is noteworthy that the clusters determined with our methods are not influenced by the functional change in E-I balance occurring in different photoperiods. This is advantegous since our analysis will also detect functional clusters within neuronal networks with altered E/I balance often found in neurological disease (e.g. epilepsy, RETT, FragileX, autism) and in the aging brain.

The results presented here show that our method offers great potential for detecting hidden functional synchronization and desynchronization in brain networks and are not limited to gene expression rhythms. Time series from other modalities, such as electrical action potential recordings, EEG recordings and fMRI recordings can also be interpreted through this new method. As such, the method may offer diagnostic or pre-diagnostic applications in medical health care.

## Materials and methods

### Ethics statement

The experiments were performed in accordance to the Dutch law on Animal welfare and approved by the Dutch government (DEC 11010).

### Bioluminescence imaging

Experimental methods and results are treated in our accompanying paper [7]. Briefly, male homozygous PER2::LUCIFERASE knock-in mice [47] were bred in the animal facility of the Leiden University Medical Center (LUMC). The animals were entrained to different photoperiods, being either summer days with 16 hours of light and 8 hours of darkness (LD 16h:8h) or to winter days with 8 hours of light and 16 hours of darkness (LD 8h:16h). The mice were entrained for at least 28 days to their respective photoperiod. Animals were sacrificed within two hours before lights off, since dissection during that period is found to least affect the SCN rhythm [48, 49].

Organotypic cultures of the SCN were prepared as described previously [7]. In brief, mice were truncated and the brain immediately dissected and placed in ice cold, low Ca^2+^ and high Mg^2+^ artificial cerebrospinal fluid (ACSF), containing (in mM): NaCl (116.4), KCl (5.4), NaH_2_PO_4_ (1.0), MgSO_4_ (0.8), CaCl_2_ (1.0), MgCl_2_ (4.0), NaHCO_3_ (23.8), D-glucose (16.7) and 5 mg/L gentamicin (Sigma Aldrich) saturated with 95% O_2_—5% CO_2_ (pH 7.4). From each animal, the hypothalamus containing the SCN was cut in 200 μm thick slices, using a VT 1000S vibrating microtome (Leica). From two consecutive coronal slices (the SCN was isolated and placed on a Millicell membrane insert (PICMORG50, Millipore). Membrane inserts were placed in a 35 mm dish, which contained 1.2 mL of Dulbecco’s Modified Eagles Medium (D7777, Sigma-Aldrich) supplemented with 10 mM HEPES-buffer (Sigma-Aldrich), 2% B-27 (Gibco), 5 U/ml penicillin and 5 *μ*g/ml streptomycin (0.1% penicillin-streptomycin, Sigma-Aldrich) and 0.2 mM D-luciferine—sodium salt (Promega), adjusted to pH 7.2 with NaOH. The dish was sealed with vacuum grease (details) and a 40 mm coverslip.

The dish containing the cultured SCN tissue were immediately transferred to a light tight and temperature controlled chamber, kept at 37 °C (Life Imaging Services, Reinach, Switzerland). The chamber was equipped with an upright microscope (BX51WIF, Olympus) with a long-working distance objective (HN10X/22, Olympus) and a cooled CCD camera (ORCA –UU-BT-1024, Hamamatsu). The bioluminescence images were obtained from two SCN cultures per experiment, with an exposure times of 29 min, resulting in one image per hour. Stage and focus position, as well as image acquisition was controlled by Image Pro Plus software (MediaCybernetics, Warrendale PA USA; StagePro plug-in, Objective Imaging, Cambridge, UK), driving a motorized stage (XY-shifting table 240, Luigs & Neumann Ratingen, Germany) and a focus control (MA-42Z, Märzhäuser, Wetzlar, Germany) both connected to an OASIS-4i Four Axis Controller.

### Data processing

A MATLAB-based (Mathworks, Natick, MA) custom-made program was used to analyze the images. An automated detection procedure identified cell-like regions of interest (ROIs) consisting of groups of pixels with luminescence intensity above the noise level. The time series from the cell-like ROIs were smoothed and the data was resampled to one data point per minute to reduce noise and increase the efficiency for subsequent analyses [50]. The cell-like ROIs were evaluated on consistency of location throughout the recording, and the smoothed signals on sustained PER2::LUC signal and circadian rhythmicity. All single cells had a minimum of three consecutive cycles, where the average peak interval was in the circadian range (20-28h).

Both raw data as well as smoothed data were tested using the mathematical method described in this paper, which did not yield significantly different results.

### Modularity for correlation matrix

We describe the redefined modularity for correlation matrices [15]. Let us consider a system with *N* cells. One can introduce a number of partitions of the *N* cells into non-overlapping sets. The different partitions will be represented by an *N*-dimensional vector $\vec{\sigma }$ where the *i*-th component *σ*_*i*_ denotes the set in which cell *i* is placed by that particular partition. Now, we introduce the modularity measure $Q(\vec{\sigma })$ which indicates the quality of a particular choice of partition $\vec{\sigma }$ measured by a high degree of inter-community connectivity and a low degree of intra-community connectivity. So-called modularity optimization algorithms look for the specific partition that maximizes the value of $Q(\vec{\sigma })$, the objective function. The latter is defined as

$$\begin{array}{c} Q\left(\vec{\sigma }\right)=\frac{1}{C_{norm}}\sum_{i,j}[C_{ij}-\left\langle C_{ij}\right\rangle ]\delta \left(\sigma_{i},\sigma_{j}\right) \end{array}$$ (1)

where 〈*C*_*ij*_〉 is a null model that needs to identify the random properties of empirical correlation matrices.

In this approach, the empirical correlation matrix is first decomposed and then reconstructed using only the eigenvalues (and eigenvectors) that are not reproduced by the random null model. Once compared with the observed spectrum of the empirical correlation matrix, the model will identify the non-random eigenvalues (by elimination). The non-random eigenvalues will be later used to generate the new filtered matrix.

### Null model for neural system

**Fig 8:**
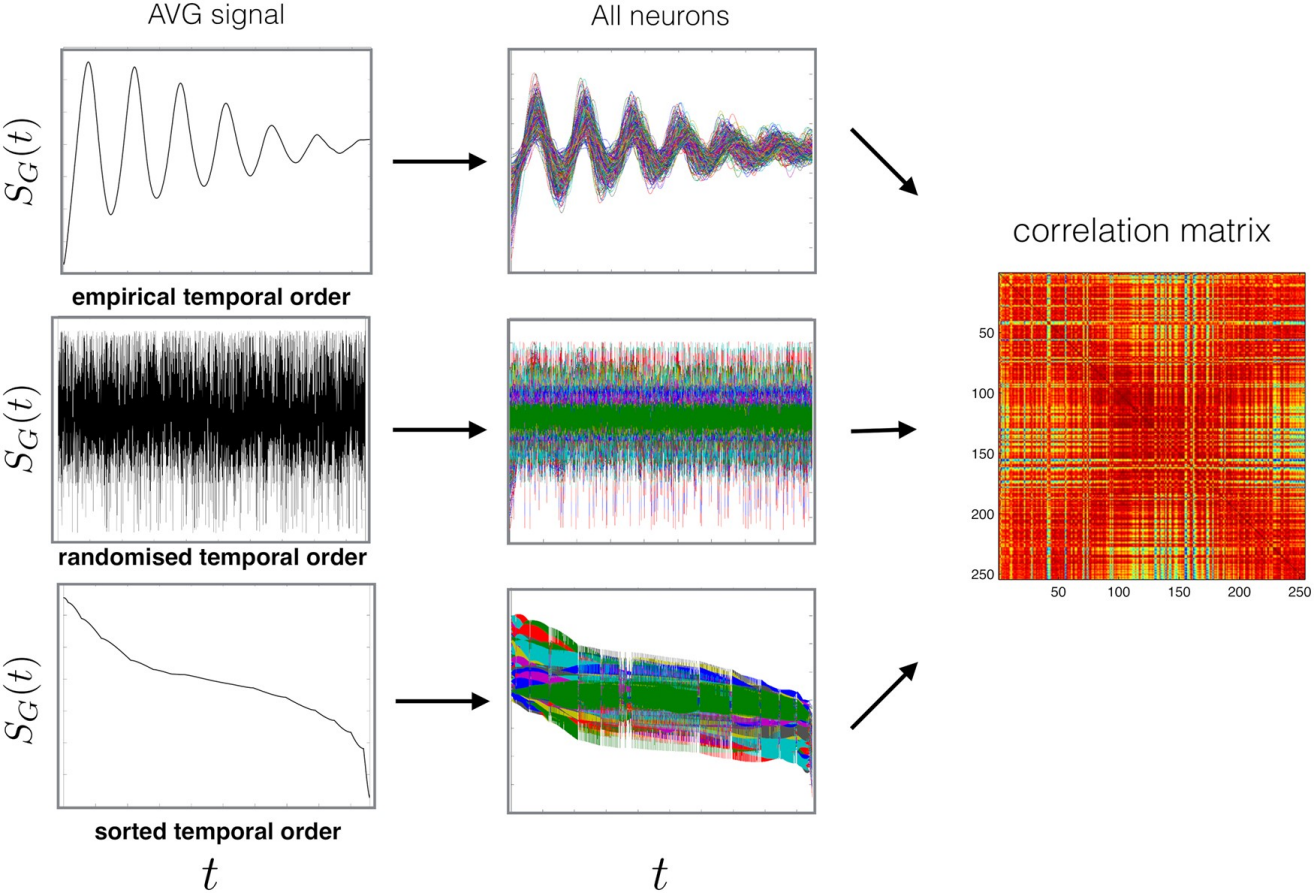
Temporal modulation. Different temporal modulation in the data result in an identical correlation matrix. A comparison between the empirical data and reorganized versions of the empirical data, highlighting the influence of a presence of a global trend on a correlation matrix. We show that by construction a Pearson correlation matrix can not differentiate between the dynamics of the global trend. Hence, the effects of different (dynamics of) global modes can be filtered at the level of the correlation matrix.

Here we proceed to the exact calculation of the null model, that will be used as a random benchmark in the modularity. The aim is to calculate the the density of eigenvalues *ρ*(λ) of a theoretical cross-correlation matrix, however, here we look at a special case of a random system with common trends. As a crucial difference with respect to a similar method introduced in [15], we do not require the unrealistic assumption that the time series are stationary. Therefore we can allow for any temporal modulation (see Fig 8), both in individual time series and in their resulting common trend [see section Materials and methods]. This is very important, given the strongly time-dependent nature of functional brain data in general, and of our time-modulated oscillating signals in particular. So, even if the calculation and interpretation of correlation matrices usually assumes stationarity, here we can statistically treat correlation matrices calculated from nonstationary data as well.

A second and related improvement takes into account the effects of common (nonstationary) trends for a system with *N* cells, and in particular the largest eigenvalue λ_*max*_. We realize that the effects of noise are inseparably coupled to those of the global trend [51], as the presence of the latter modifies and left-shifts the density of eigenvalues that we would otherwise observe in presence of noise only. So we do not simply superimpose the two effects as in [15]; on the contrary, we calculate the modification of the random bulk exactly, given the system’s empirical λ_*max*_. In particular, we calculate the shifted value of an original Wishart matrix [15] to find

$$\begin{array}{c} \lambda_{\pm }=(1-\frac{\lambda_{max}}{N}){\left(1\pm \frac{1}{\sqrt{Q}}\right)}^{2} \end{array}$$ (2)

where *Q* = *T*/*N* is the ratio between the number of time steps in the data *T* and the number of cells *N*. Fig 2 shows both the modified and unmodified spectral densities. It also shows that taking the left-shift of the random bulk into account is very important, as it unveils informative empirical eigenvalues that would otherwise be classified as consistent with the random spectrum and hence discarded.

### Community detection algorithm

We employ a popular community-detection technique that take the filtered correlation matrix as input and return the best partition of the system into functional modules [23, 52]. In this set-up the algorithm is clustering positive dependencies within the clusters and expelling negative dependencies outside. The optimized partition, which maximizes the modularity Eq 1, is considered the binary signature of the system.

We should stress that while standard community-detection methods are based on null models that are justified only for networks, but not for time series, our method builds on the appropriate null model described above and calculated exactly in the first step.

**Fig 9:**
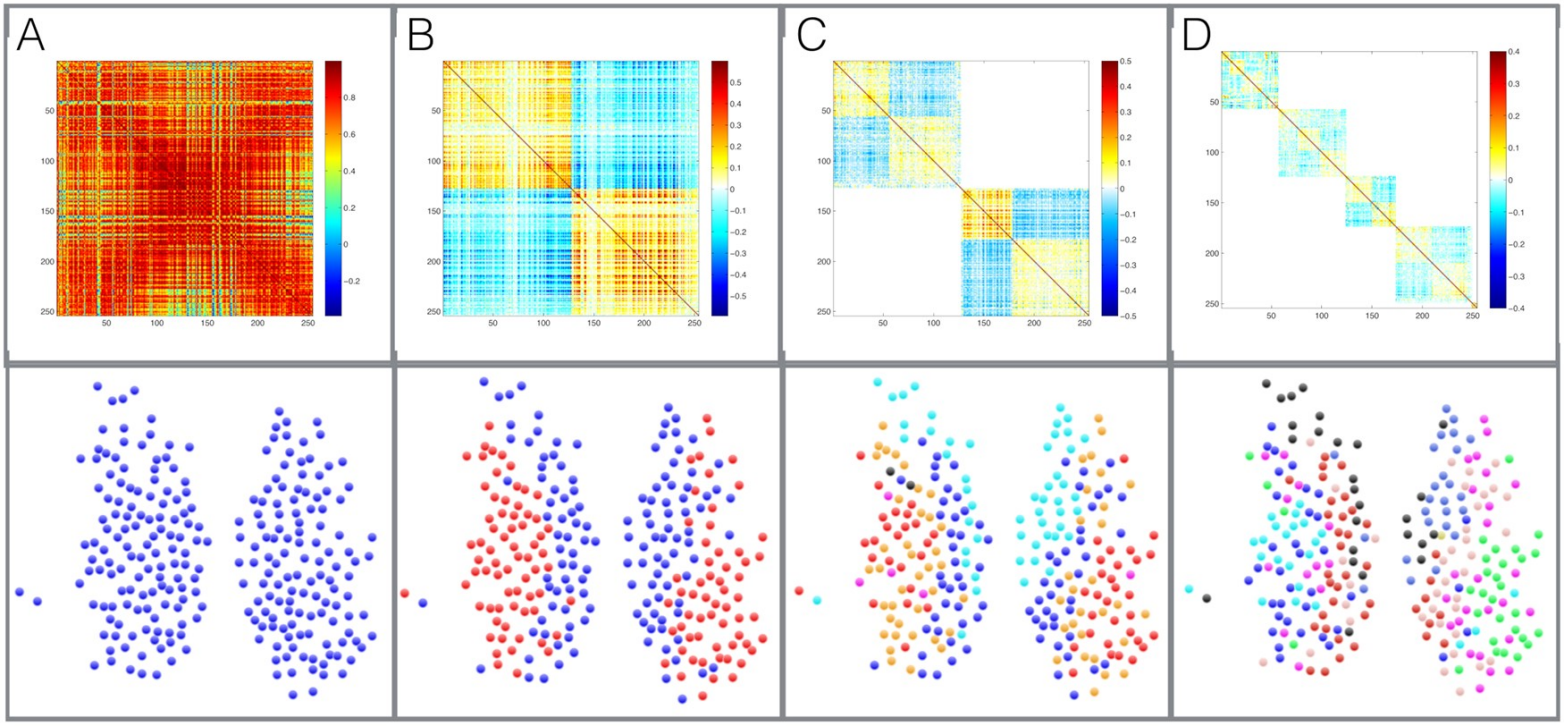
The hierarchical community structure of the SCN as resolved by our method. The community structure detected by different recursive runs of the method (A,B,C,D). In the bottom panels are the partitions detected, where each community is marked with a different colour. In the top panels are the corresponding resolved filtered correlation matrices (for each run) displaying the resolved structure as a block matrix.

The use of a correct null model allows for a recursive analysis of specific time series in the data, i.e. analyze different hierarchical levels of the community structure. Here, unlike in the analysis of a network topology, further analyzing the communities for the detection of sub-clusters is not acting on missing information (ignoring inter-clusters links). We take the original time series of each cluster and construct a new correlation matrix, this matrix will then be filtered and analyzed with the same approach. In Fig 9 we present an hierarchical community structure of an SCN sample as resolved by our method. In this study we only explore the first partition, since the data contains a limited number of cells, which makes the next partitions unreliable. However, this feature marks a great potential for future data sets and studies.
